# Vascular Inflammation in Mouse Models of Systemic Lupus Erythematosus

**DOI:** 10.3389/fcvm.2022.767450

**Published:** 2022-03-28

**Authors:** Holly Ryan, Laurence Morel, Erika Moore

**Affiliations:** ^1^J. Crayton Pruitt Family Department of Biomedical Engineering, University of Florida, Gainesville, FL, United States; ^2^Department of Pathology, Immunology and Laboratory Medicine, University of Florida, Gainesville, FL, United States; ^3^Department of Materials Science and Engineering, University of Florida, Gainesville, FL, United States

**Keywords:** vascular inflammation, systemic lupus erythematosus, *in vitro* disease modeling, autoimmune disease, mouse models, cardiovascular diseases

## Abstract

Vascular inflammation mediated by overly activated immune cells is a significant cause of morbidity and mortality in systemic lupus erythematosus (SLE). Several mouse models to study the pathogenesis of SLE are currently in use, many of which have different mechanisms of pathogenesis. The diversity of these models allows interrogation of different aspects of the disease pathogenesis. To better determine the mechanisms by which vascular inflammation occurs in SLE, and to assist future researchers in choosing the most appropriate mouse models to study cardiovascular complications in SLE, we suggest that direct comparisons of vascular inflammation should be conducted among different murine SLE models. We also propose the use of *in vitro* vascular assays to further investigate vascular inflammation processes prevalent among different murine SLE models.

## Introduction

Systemic lupus erythematosus (SLE) is an autoimmune disease characterized by production of antibodies that react to self-antigens such as DNA and complement components (1). Vasculitis and progression to cardiovascular diseases (CVD) are prevalent and significant contributors to mortality in SLE (2). Although the pathogenesis of CVD in SLE is not fully understood, it is known that abnormalities in immune cells are heavily implicated. Many immune cells, such as T cells and monocytes, are overactive in SLE, causing chronic inflammation and widespread tissue damage, including damage to the heart and vasculature (3). Significant advances toward understanding SLE pathogenesis have been made in the past 60 years due to the use of mouse models that recapitulate certain features of the human disease. However, few studies have addressed cardiovascular complications. Here, we propose that an in-depth systematic characterization of CVD phenotypes in mouse models of SLE in relationship with immune alterations will improve the understanding of the pathogenesis of CVD in SLE. Additionally, we argue that application of *in vitro* biomaterial models will also contribute to increased understanding of CVD pathogenesis in SLE.

## CVD in Human SLE

One of the most serious complications of SLE is CVD, which is a leading cause of death five years past diagnosis (2, 4). With an incidence ranging from 31 to 70% (5–7), CVD presents in SLE patients with diverse manifestations including pericarditis, myocarditis, valvular disease, atherosclerosis, thrombosis, and arrhythmias. This clinical heterogeneity likely reflects a complex etiology as well as the contribution of multiple risk factors. Widespread use of imaging tools has revealed a high frequency of microvascular impairment and myocarditis in SLE patients (6, 8–11), the majority of which do not lead to clinical presentation (12–14). Perfusion abnormalities have also been detected by single-photon emission computerized tomography imaging in 88% of SLE patients, two thirds of which had negative coronary angiograms (15). These results are in agreement with the reduction of myocardial coronary flow reserve on MRI studies found in 44% of SLE patients with angina and a normal angiogram (15). These findings suggest that coronary microvascular dysfunction, which has emerged as a mechanism of myocardial ischemia, heart failure, and arrhythmias distinct from obstructive atherosclerosis, is a common feature in SLE patients, but that the dysfunction is difficult to assess, and probably underdiagnosed.

In addition to coronary effects, vascular inflammation in SLE has further implications throughout the rest of the body. About 11–36% of SLE patients experience vasculitis, which may affect small, medium, or large vessels, causing damage in the integumentary, neurological, digestive, respiratory, and urinary systems (16). Vasculitis is thought to be mediated by immune complex deposition along vessel walls, as well as by direct destruction of vessel components by anti-endothelial cell autoantibodies (3, 16). The binding of immobilized antibodies and immune complexes by innate immune cells such as monocytes sets off an inflammatory response.

Endothelial function deteriorates with increased activity of type-1 interferons (IFNs), an important family of inflammatory cytokines that are upregulated in SLE (3). Endothelial dysfunction is thought to contribute to the dramatically increased risk of hypertension in SLE patients (17). SLE patients are also at risk of developing atherosclerosis and suffering ischemic events such as ischemic stroke or myocardial infarction (3). Atherosclerosis in SLE may be associated with vasculitis since damage to endothelial cells (ECs) is known to lead to CVD (3). New or worsening atherosclerosis occurs in 10% of SLE patients per year, although the precise mechanism by which it occurs is not fully understood (18).

Further study into the mechanisms driving SLE vasculitis is needed to identify targets for treating this serious co-morbidity. Since SLE is widely studied using mouse models, in this review we describe the cardiovascular manifestations of disease in several of the most common SLE mouse models. Unfortunately, most SLE mouse models do not develop cardiovascular complications comparable to those experienced by SLE patients. For this reason, we also propose future work that leverages biomaterial model systems to assist in identifying processes relevant to human SLE-associated CVD.

## CVD in SLE Mouse Models

Several spontaneous and induced mouse models of SLE have been developed since the 1960s, with overlapping but distinct mechanisms of disease, allowing for the study of isolated disease processes. We refer the reader to recent review articles detailing the pathogeneses of several of these models (19–21), as well as to a review article focusing on the development of myocardial infarction or hypertension in several of these models (22). Table 1 gives a summary of commonly studied CVD manifestations observed in SLE mouse models.

**Table 1:** Cardiovascular manifestations associated with different SLE mouse models.

**Mouse models**		**Cardiovascular manifestations**	**References**
Induced models	Pristane-induced lupus	Pulmonary vasculitis, hypertension	(19, 20, 23–26)
	Imiquimod-induced lupus	Hypertension, increased left ventricular weight	(27, 28)
NZB/NZW F1		Cardiac inflammation, hypertension	(19, 22, 29–38)
NZM strains		Thrombosis in response to endothelial injury, impaired endothelial cell differentiation	(19, 20, 39, 40)
B6.NZM2410.*Sle1.Sle2.Sle3* triple congenic		Enhanced atherosclerosis susceptibility in LDLr or ApoE KO mice	(41–44)
Fas mutation (*gld*.apoE^−/−^, B6.MRL-Fas*^*lpr*^*, and MRL/Fas*^*lpr*^*)		Increased risk of atherosclerosis and myocardial infarction in LDLr or ApoE KO mice	(19, 22, 38, 45–54)
Yaa-carrying strains	BXSB	Increased risk of myocardial infarction	(19, 22)
	(NZW/BXSB) F1	Coronary artery disease from anti-phospholipid syndrome and microthrombi, increased risk of myocardial infarction	(22, 55, 56)
	B6.Nba2.*Yaa*	Increased atherosclerosis susceptibility	(57)

Although there have been few studies comparing microvascular inflammation qualitatively and quantitatively among different SLE mouse models, there are several metrics summarized in Table 2 by which SLE-associated CVD can be assessed. Overt atherosclerosis can be observed as plaques that are visible by histology in some mouse models such as apolipoprotein E (ApoE) knockouts. Production of reactive oxygen species and associated enzymes in disease vasculature can also be measured using histology in NZB/NZW F1 mice and in some imiquimod-treated models (27, 34, 37). Some strains including MRL/Fas^*lpr*^, BXSB, and (NZW/BXSB) F1, may also develop myocardial infarctions (55). NZB/NZW F1 mice and some induced models develop hypertension, which may be used as another measure of CVD progression (23, 25, 27, 32–36, 44).

**Table 2:** Experimental measures of CVD in SLE mouse models.

**Metric**	**Results when CVD is present**	**Mouse model**	**References**
Tissue lesions (histology)	Visible atherosclerotic lesions	Mice susceptible to atherosclerosis such as ApoE or LDLr knockouts or hybrids	(26, 39, 41, 43, 44, 46, 48, 50, 52, 57)
	Myocardial infarction	(NZW/BXSB) F1, MRL/Fas*^*lpr*^*, BXSB	(40, 50)
Hypertension	Increased systolic blood pressure and/or mean arterial pressure	Induced models (pristane or imiquimod); NZB/NZW F1	(23, 25, 27, 31–36, 44)
Endothelial dysfunction	Impaired endothelium-dependent vasorelaxation	Can be used in any model but frequently used in mice that do not develop atherosclerosis, such as NZB/NZW F1; also frequently used in induced models	(23–25, 27–30, 34–40, 48, 53, 54)
	Reduced EPC proliferation/differentiation	Can be used in most models, including pristane or imiquimod-treated mice, NZB/NZW F1, NZM2328, *gld*.apoE^−/−^, and MRL/Fas*^*lpr*^*	(24, 28, 30, 38–40, 50, 53)

Since many SLE mouse models do not display overt atherosclerosis and hypertension in the same manner as humans, histological, and blood pressure studies are often insufficient for assessing CVD progression. In these cases, vascular disease can be measured by functional studies in which isolated arteries are forced to contract, and then are exposed to various vasodilators to observe the extent of vasorelaxation (23–25, 27–30, 34, 35, 37–40). This type of study can be done on any mouse model. Another metric of CVD progression is the proliferation of endothelial progenitor cells (EPCs) and their ability to differentiate into mature ECs. EPCs from bone marrow, spleen, and the peripheral circulation of SLE mice often display decreased proliferation and differentiation compared to wild-type mice, suggesting a role for impaired endothelial turnover in SLE-related CVD (24, 30, 38–40, 50, 53). The various methods commonly employed to measure CVD in SLE mouse models are summarized in Table 2.

The following sections describe how these metrics have been used to investigate CVD in mouse models of SLE.

### Tissue Lesions Detected by Histology

#### Atherosclerosis

A very common method to assess the extent of CVD in an animal model is to observe vessel micrographs for the presence of atherosclerotic lesions; however, there are few SLE mouse strains that develop atherosclerosis naturally. For this reason, pro-atherogenic mouse models such as ApoE knockouts are often used to investigate how certain cytokines associated with SLE, such as type I IFNs, may contribute to the development of vascular lesions (26). Increasing type I IFN levels by infecting ApoE^−/−^ mice with IFNα-expressing adenovirus has been shown to increase vascular lesions, while the opposite was seen by knocking out of the IFNα receptor (IFNAR) (39). Knockout of ApoE or low density liproprotein receptor (LDLr) has also been applied to various SLE -specific mouse models to demonstrate the role of SLE pathways in worsening plaque formation in mice that are already prone to atherosclerosis (41, 43, 44, 50, 57). For example, ApoE^−/−^ mice manifest histologically observable vascular lesions upon treatment with pristane, an inflammation-inducing hydrocarbon that promotes type I IFN production (26). The use of type I IFN-increasing agents in mice that are susceptible to atherosclerosis provides evidence that the increase in type I IFNs seen in SLE patients is a major driver behind CVD in these patients.

Another mouse model that has been used in combination with atherosclerosis-prone models is the B6.NZM2410.*Sle1.Sle2.Sle3*, or triple congenic, mouse. This mouse has three NZM2410-derived-SLE susceptibility loci on a C57BL/6 genetic background. Furthermore, this phenotype maps, at least in part, to the overexpression of the lupus susceptibility gene Pbx1-d, which impairs regulatory T cells (43). Triple congenic mice do not develop atherosclerosis spontaneously, but they have been used as bone marrow donors in chimera studies, with atherosclerosis-prone strains as recipients. Chimeras of LDLr^−/−^ (44) or LDLr^−/−^Rag^−/−^ mice (41) with bone marrow from triple congenic mice have shown increased atherosclerosis compared to chimeras with bone marrow from control C57BL/6 mice (42). Mutations in Fas or the Fas ligand (FasL), which disrupt apoptosis, have also been introduced into atherogenic mouse models to simulate the CVD effects of SLE. For example, the FasL mutation in *gld*.*apoE*^−/−^ mice causes glomerular lesions such as those seen in SLE as well as the vascular lesions typical for ApoE^−/−^ mice (50).

#### Myocardial Infarction

Although thrombosis and myocardial infarction occur frequently in human SLE patients, there are relatively few SLE mouse strains that develop these complications. Some of the most commonly used mouse strains to study thrombosis and myocardial infarction in SLE are mice with mutations in Fas or FasL, or with overexpression of Toll-like receptor 7 (TLR7).

Fas is a membrane-bound receptor that triggers apoptosis. The Fas/FasL pathway is especially important for inducing apoptosis in activated lymphocytes after infection has been cleared (58). Mutations in Fas or FasL are present in *lpr* and *gld* mice, respectively, and lead to the development of a lupus-like autoimmune pathology. Mice with these mutations often have more obvious CVD than other SLE strains. As they age, MRL/Fas^*lpr*^ mice develop necrotizing polyarteritis with rare thrombotic occlusion (51, 52). MRL/Fas^*lpr*^ males tend to develop age-dependent myocardial infarction (22). A recent study examining multiple organs has also shown vascular and perivascular leukocyte infiltrations increased as the mice aged and developed autoimmune pathology (48). These observations suggest that the vascular inflammation and increased risk of myocardial infarction in SLE may be due to active lymphocytes that have failed to receive a normal cell death signal.

The BXSB mouse has a translocation of TLR7 from the X chromosome to the Y chromosome, termed *Yaa*, causing males to develop SLE-like symptoms due to overactivation of the type I IFN pathway, a downstream effect of TLR7 signaling (19). BXSB mice may have increased risk of myocardial infarction, but the risk is lower than for other strains such as (NZW/BXSB) F1 males or MRL/Fas^*lpr*^ mice (22, 55). (NZW/BXSB) F1 male mice, which are the offspring of the cross between NZW females and BXSB males, have a similar course of disease to that of BXSB males, but with more clearly prevalent coronary vascular disease and myocardial infarction (22). Myocardial infarction in these mice may be due to small coronary artery disease as well as vascular lesions caused by anti-phospholipid autoantibodies (55).

### Hypertension

The exact pathogenesis of hypertension in human SLE is not well-understood, but is thought to be related to some combination of endothelial dysfunction, kidney damage, abnormalities in the renin-angiotensin-aldosterone system, dysautonomia, and increased endothelin-1 (17). The degree of hypertension in mice may be measured by tail cuff for systolic blood pressure (33, 34) or by catheterization for mean arterial pressure (35, 36). The main mouse models generally used to study SLE hypertension are pristane-induced models and the NZB/NZW F1 strain. C57BL/6 and BALB/c mice develop increased arterial pressure when treated with pristane, suggesting that that an increase in type I IFN contributes to hypertension (23, 25).

NZB/NZW F1 mice develop spontaneous hypertension that may be avoided by therapeutic intervention to curtail SLE development (32, 33), although one study showed that treating the SLE-like glomerular damage and inflammation seen in this mouse did not decrease blood pressure (33). For this reason, it has been suggested that hypertension and kidney disease in this model are not directly related (33). Hypertension in NZB/NZW F1 mice may be attributable to a variety of influences including increased sensitivity to angiotensin II (31). Inhibition of angiotensin II by the angiotensin-converting enzyme (ACE) inhibitor captopril has been shown to downregulate expression of the type I IFN regulator *Ifr7* (59), so it is possible that the increased angiotensin II sensitivity in NZB/NZW F1 mice causes hypertension mainly *via* increase in type I IFNs.

### Endothelial Dysfunction

#### Vasorelaxation

Another common method of measuring vascular dysfunction is through vasorelaxation studies. These studies are useful because they can be done even if a particular strain of mouse is not prone to developing atherosclerosis or hypertension. In these studies, excised vessels are contracted using a vasoconstrictive agent such as phenylephrine (PE) or U-46619, and then exposed to different vasodilators. Acetylcholine (Ach) causes vasorelaxation in an endothelium-dependent manner by stimulating nitric oxide production, while sodium nitroprusside induces vasorelaxation in an endothelium-independent manner. Vessel response to Ach can be compared to the response to sodium nitroprusside to determine whether impaired vasorelaxation is due to endothelial dysfunction.

Vasorelaxation in response to Ach is impaired in many models of SLE that have type I IFN as a major driver of disease, including ApoE^−/−^ mice exposed to IFNα-expressing adenovirus (39), pristane or imiquimod-treated mice (23–25, 27, 28), NZB/NZW F1 mice (35, 37, 38), and NZM2328 mice (39). Generally, these mice do not display impaired vasorelaxation in response to sodium nitroprusside, which induces vasorelaxation by acting directly on the vascular smooth muscle. These findings demonstrate that models with high levels of type I IFN experience impaired vasorelaxation due to endothelial dysfunction, which is also a common complication in human SLE.

Findings about vasorelaxation in mice with Fas or FasL mutations are variable. Compared to MRL/MpJ, a common control for MRL/Fas^*lpr*^ mice, MRL/Fas^*lpr*^ mice have decreased vasorelaxation in response to Ach (53, 54); however, B6.MRL-Fas^*lpr*^ mice have increased vasorelaxation in response to Ach and to sodium nitroprusside compared to C57BL/6 controls, even in the setting of high proteinuria, which indicates advanced disease (38). B6.MRL-Fas^*lpr*^ mice experience less severe disease than MRL/Fas^*lpr*^ mice (47), so the difference in vasorelaxation ability suggests that the *lpr* mutation alone is not sufficient to cause endothelial dysfunction. In addition, B6.MRL-Fas^*lpr*^ mice do not overproduce type I IFN-regulated genes (38), which seem to be responsible for endothelial dysfunction in most of the other models. For this reason, although B6.MRL-Fas^*lpr*^ mice are useful for modeling SLE in other respects, they may not be appropriate to use in studies of SLE-related endothelial dysfunction.

#### EPC Proliferation/Differentiation

Another means of measuring endothelial dysfunction is to investigate the proliferation and differentiation potential of EPCs. In healthy vasculature, EPCs replace old ECs to maintain the integrity and functionality of the endothelium (60). If EPCs are reduced, or if they are unable to differentiate into mature ECs, the turnover of the endothelium is impaired, resulting in vascular disease. Studies on the proliferation and differentiation potential of EPCs have been done on a wide variety of SLE mouse models. EPC differentiation was reduced in pristane- or imiquimod-treated mice (24, 28), in NZB/NZW F1 mice (38), and in ApoE^−/−^ mice exposed to IFNα-expressing adenovirus (39). In aged NZM2328 mice, IFNAR deletion increased the numbers of both bone marrow and circulating EPCs; and in both young and aged female mice, EPCs had increased differentiation ability in the absence of IFNAR (39). These observations corroborate similar findings from vasorelaxation studies, demonstrating that the type I IFN pathway is involved in SLE-related endothelial dysfunction. On the other hand, B6.MRL-Fas^*lpr*^ mice do not display reduced numbers of EPCs in the bone marrow, or decreased EPC differentiation (38). Disease is not primarily mediated by type I IFNs in B6.MRL-Fas^*lpr*^ mice, so this result is unsurprising.

## Future Directions

Several studies have investigated cardiovascular outcomes in individual SLE mouse models compared to control mice, but thus far very few studies have directly compared cardiovascular outcomes among different SLE mouse models.

Transcriptional signatures between NZB/NZW F1, NZW/BXSB, and NZM2410 mice have been compared to one another and to those of human SLE patients to identify common pathways (61, 62). These studies identified the STAT3- and IL-36A pathways shared between all models. Differences between strains were also seen for some genes, such as increased mitochondrial dysfunction signatures in NZB/W F1 and NZM2410, but not in NZW/BXSB, suggesting that NZB/W F1 and NZM2410 strains have more oxidative stress and so may better simulate human conditions for any future studies on mitochondria in SLE (61). Importantly, such advances have correlated with significant outcomes seen in profiling lupus nephritis in human patients *via* single cell sequencing (scRNAseq) (63, 64). The identification of gene expression pathways that are shared not only among different mouse models but also between mice and humans is an important step toward development of more effective therapeutics.

While the studies mentioned above used histology to study the extent of nephritis, which is a well-known pathology caused by SLE, histological studies of atherosclerosis and other cardiovascular complications of SLE are more challenging. Mice do not develop atherosclerosis with the same pathogenesis as humans; for example, lesions develop in different vessels and have different histological features (65). Some of these changes may be due to the size and hemodynamic properties of mouse vasculature (65). In addition, as discussed in the previous sections, most murine SLE models do not present with overt cardiovascular symptoms. As modeled for lupus nephritis, we suggest that conducting head-to-head comparisons of cardiovascular complications, such as Ach-mediated vasorelaxation, among different SLE mouse models would contribute greatly toward a better understanding of CVD progression in human SLE patients. Since different SLE mouse models have different, well-characterized mechanisms of disease, any differences in endothelial dysfunction among these models could shed more light on the pathways leading to CVD in SLE. This could, in turn, help in identification of potential new treatment options for use in the clinic. Direct comparisons of CVD development among different SLE models would also provide detailed information to assist researchers in the selection of the best mouse models to use in future studies of different aspects of SLE progression.

To better model the contribution of SLE to vascular inflammation in humans, we also propose that *in vitro* studies incorporating immune cells and ECs from different murine SLE models would be useful. Such studies would allow inflammatory pathways common between human and murine SLE cells to be investigated without potential effects from hemodynamic differences. Many types of *in vitro* platforms such as microfluidics devices and transwell assays allow for the study of interactions between diseased inflammatory cells, such as T cells or monocytes, with ECs (66). For example, in one study conducted to observe changes in angiogenesis in the setting of glioblastoma, ECs displayed increased sprouting when co-cultured with tumor-associated macrophages than with unstimulated macrophages (67). We suggest that similar studies using immune cells from SLE mice may also be informative. Since both the immune cells and the ECs of SLE mice tend to be abnormal, researchers may mix and match which cell types come from SLE mice and which come from controls. This flexibility would assist in differentiating whether endothelial dysfunction is due to an intrinsic pathology in ECs themselves, or to their interaction with abnormal immune cells. *In vitro* cultures can also be performed to study the effect of lupus serum on ECs from control mice; for example, on EC production of reactive oxygen species (46).

Currently, some of the most commonly used *in vitro* assays for CVD studies in SLE mouse models are EPC differentiation cultures, since endothelial dysfunction arises when EPCs are not able to mature and replace old ECs (26, 30, 38, 39). In addition to EPC differentiation, EPC function can also be assessed *via* various assays for adhesion and aging (26). Mature EC activity can be assessed through migration assays in which they are allowed to grow through Matrigel and form vascular tubules (26). These types of studies are useful for identifying abnormalities intrinsic to the ECs themselves.

**Figure 1:**
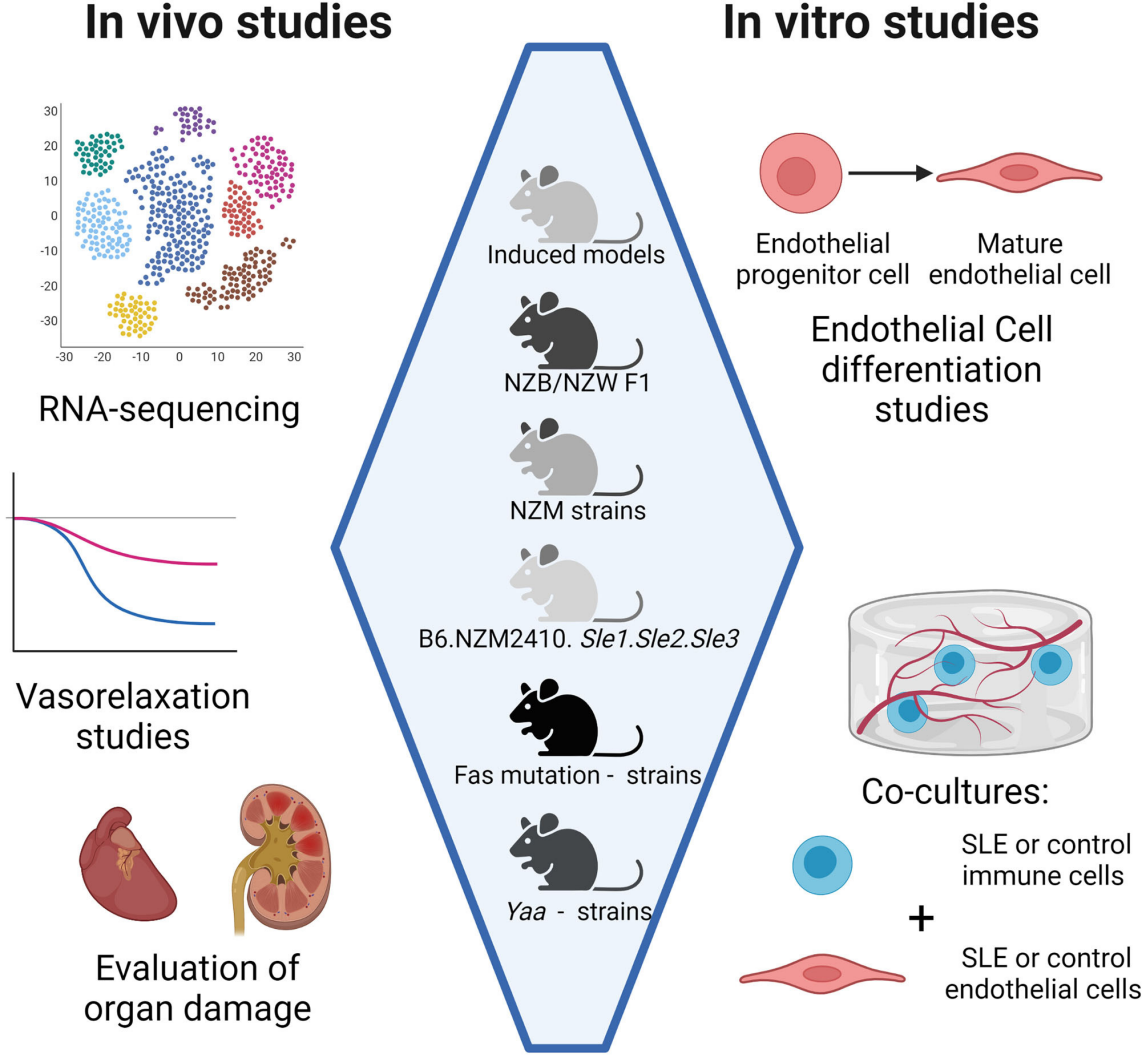
Comparison of CVD development in different SLE mouse models. Head-to-head comparisons among mouse models may be done *in vivo, ex vivo*, and *in vitro*. *In vivo* studies include RNA sequencing for expression of inflammatory genes and histology on organs such as the heart for characterization of inflammatory infiltrates. *Ex vivo* studies include vasorelaxation studies on arteries. *In vitro* studies include co-cultures of immune cells and endothelial cells, both of which can be harvested from SLE mouse models. Co-cultures may be indirect, such as in transwell systems, or direct, such as co-encapsulation studies where all cell types are embedded in a hydrogel. Created with BioRender.com.

A schematic summarizing studies that may be used to perform comparisons of vascular inflammation among different SLE mouse models is shown in Figure 1. As mentioned previously, direct comparisons between kidney gene expression in NZB/NZW F1, NZW/BXSB, and NZM2410 models have been done already (61). We propose that it may be beneficial to include additional models in future studies, such as Fas mutation-carrying strains like *gld*.apoE^−/−^, B6.MRL-Fas^*lpr*^, or MRL/Fas^*lpr*^, since the disease phenotype in these strains is not mediated by an increase in type I IFN as it is in the other strains. A comparison of vasorelaxation curves as Ach is added to a contracted section of artery could also be made among different models, as could a comparison of histological sections showing the extent of SLE progression and atherosclerosis. For *in vitro* studies, EPC differentiation assays and EC functional assays would help elucidate differences in endothelial dysfunction among different models at the cellular level. Co-culturing ECs with immune cells from different SLE models, or ECs from different SLE models with immune cells from control mice, could also indicate whether endothelial dysfunction is driven primarily by abnormalities in ECs or by abnormalities in immune cells in different models.

We propose that multiple-mouse-model studies of vascular inflammation would contribute greater understanding of the pathogenesis of CVD in human SLE. To aid researchers in the study of cardiovascular dysfunction in SLE, tendencies toward CVD in multiple different murine models of SLE should be compared head-to-head through a variety of *in vitro* and *in vivo* experiments. Comparison of models with different driving mechanisms of disease will help elucidate underlying pathways behind CVD in SLE.

## Data Availability Statement

The original contributions presented in the study are included in the article/supplementary material, further inquiries can be directed to the corresponding author/s.

## Author Contributions

All authors listed have made a substantial, direct, and intellectual contribution to the work and approved it for publication.

## Funding

The authors gratefully acknowledged funding from the National Institutes of Health NCATS 1KL2TROO1429 (EM), the Rhines Rising Star Larry Hench Professorship (EM), and the FIU/UF Collaborative Grant F020122 (HR/EM).

## Conflict of Interest

The authors declare that the research was conducted in the absence of any commercial or financial relationships that could be construed as a potential conflict of interest.

## Publisher's Note

All claims expressed in this article are solely those of the authors and do not necessarily represent those of their affiliated organizations, or those of the publisher, the editors and the reviewers. Any product that may be evaluated in this article, or claim that may be made by its manufacturer, is not guaranteed or endorsed by the publisher.
